# Tumor‐infiltrating FoxP3+ T cells are associated with poor prognosis in oral squamous cell carcinoma

**DOI:** 10.1002/cre2.477

**Published:** 2021-07-28

**Authors:** Tomio Hayashi, Kazuhiro Yoshikawa, Susumu Suzuki, Masahiko Gosho, Ryuzo Ueda, Yoshiaki Kazaoka

**Affiliations:** ^1^ Department of Oral and Maxillofacial Surgery, Graduate School of Medicine Aichi Medical University Nagakute Japan; ^2^ Research Creation Support Center Aichi Medical University Nagakute Japan; ^3^ Department of Tumor Immunology, School of Medicine Aichi Medical University Nagakute Japan; ^4^ Department of Biostatistics, Faculty of Medicine University of Tsukuba Tsukuba Japan

**Keywords:** Forkhead box P3, human papillomavirus, oral squamous cell carcinoma, regulatory T cells

## Abstract

**Objectives:**

Squamous cell carcinoma is the most common malignancy in the oral cavity. Moreover, human papillomavirus (HPV) infection has been recently implicated in the onset of oral squamous cell carcinoma (OSCC). Regulatory T cells (Tregs) are Forkhead box P3 (FoxP3) positive and are normally involved in the mechanism by which organisms escape attacks from their own immune system; however, in tumors, these cells are known to suppress antitumor immunity and block the attack against tumors. The present study evaluated the associations of the number of Tregs and HPV infection with prognoses in patients with OSCC.

**Material and methods:**

Samples from 106 patients diagnosed with OSCC were evaluated by immunohistochemical staining for the identification of FoxP3+ Tregs and HPV. The relationship between the observed number of Foxp3‐positive cells, the presence/absence of HPV infection and associations with clinicopathological indicators were analyzed.

**Results:**

Tissues were classified into high (High) and low (Low) Treg count groups, with 69 patients classified as High and 37 classified as Low. The prognoses were significantly better in the Low group compared with the High group (*p* = 0.04). FoxP3 expression may have had some effect on nodal metastases (*p* = 0.09). HPV antigens were detected in 65 patients, but there were no significant associations with prognosis (*p* = 0.34). HPV‐infected tumors were more common in the gums and tongues than in the lips, cheeks, and floor of the mouth (*p* = 0.05).

**Conclusions:**

These results indicate that Tregs in tumor sites are associated with worsened prognoses of patients with OSCC and suggest potential therapies targeting Tregs in OSCC.

## 1 INTRODUCTION

Head and neck cancers represent the sixth‐most frequent cancer with approximately 500,000–600,000 patients being affected each year worldwide (Leemans et al., 2011). Approximately 50% of patients have cervical lymph node metastases at the time of diagnosis, and the prognosis of these metastatic patients is poor (Kowalski et al., 2000). For treatment outcomes, there have been no major changes in overall patient survival, and the 5‐year survival remains only approximately 64.4% (Zanoni et al., 2019).

Smoking and alcohol consumption are two major risk factors for the onset of head and neck cancer, and their synergistic effects have also been reported (Hashibe et al., 2009). In recent years, human papillomavirus (HPV) has attracted increasing attention as a risk factor in addition to those already known, and in particular, the association between oropharyngeal cancer and HPV infection has been strongly suggested (Gillison, 2000). Meanwhile, among head and neck cancers, the clinical relevance of HPV infection in oral squamous cell carcinoma (OSCC) remains unclear (Lai et al., 2017). Despite developments in the treatments for head and neck cancer and advances in elucidating the mechanisms of carcinogenesis, prognoses remain unsatisfactory (Leemans et al., 2011), and the 5‐year disease‐free survival remains poor due to uncontrolled advanced cancer, local recurrences, and distant metastases. Thus, early diagnosis and treatment initiation and the development of effective therapies other than conventional surgery, chemotherapy, and radiotherapy are essential. To achieve this, the immune responses at the local sites of head and neck cancer must be clarified and our understanding of the molecular biology of cancer must be improved.

Recently, the analysis of immune defense mechanisms in living organisms has progressed, and in particular, the presence of mechanisms that suppress the activity of lymphocytes that are reactive with self‐molecules (immune checkpoints) and lymphocytes that suppress the action of activated lymphocytes [regulatory T cells (Tregs)] has become evident. Key immune checkpoint molecules include programmed death‐1 (PD‐1) discovered by Ishida et al. (1992) and its ligands programmed death‐ligand 1 (PD‐L1) and programmed death‐ligand 2 (PD‐L2) (Freeman et al., 2000; Latchman et al., 2001). PD‐1 is expressed on activated T lymphocytes, and the mechanism by which the activity of activated T cells is suppressed involves its binding to PD‐L1 expressed on target cells. This mechanism is primarily an intrinsic immunosuppressive system serving as a defense mechanism from T lymphocytes that recognize autoantigens and attack autologous cells; however, this system also prevents activated T lymphocytes from attacking autologous tissue‐derived cancer cells.

Moreover, Tregs were discovered as a group of cells with self‐protective roles in suppressing the attack on autoantigens, through experiments in which T cells from the spleens of mice were transplanted into nude mice; the removal of CD25+ cells from CD25+/CD4+ T cells prior to transplantation resulted in the development of various autoimmune diseases, whereas the transplantation of CD25+/CD4+ T cells suppressed the development of autoimmune diseases (Sakaguchi et al., 1995). Tregs play an important role in immune homeostasis by maintaining immune self‐tolerance via the suppression of excessive immune responses. In 2003, it became clear that the transcription factor Forkhead box P3 (FoxP3) is not only a specific marker for Tregs but also a master regulator of development and functional activity. In addition, FoxP3 is involved in the immunosuppressive function, development, and differentiation of Tregs (Fontenot et al., 2017; Hori et al., 2017; Khattri et al., 2003). Thereafter, the role of Tregs in various immune responses were analyzed in detail, and it was revealed that Tregs play an important role not only in autoimmune diseases but also in transplantation immunity, allergic reactions, tumor immunity, and even in pregnancies by suppressing the immune response (Nishikawa & Sakaguchi, 2010; Samstein et al., 2012; Wing & Sakaguchi, 2010).

In this study, the presence of FoxP3+ Tregs locally in head and neck cancer, particularly in OSCC, was evaluated using immunohistochemical staining, and the association with clinicopathological indices was clarified to elucidate some localized cancer immune responses. The objective was to discover findings that can lead to the development of new therapies to improve survival prognoses. In addition, we aimed to clarify part of the immune responses in localized areas of virus‐infected cancers by determining the survival prognoses in patients with OSCC with and without HPV infection and the associations between the presence of FoxP3+ Tregs and clinicopathological indicators in HPV‐positive OSCC that have not yet been reported.

## 2 MATERIALS AND METHODS

### 2.1 Patients

This study was a retrospective analysis of 106 patients who were assessed at Aichi Medical University Graduate School of Medicine Oral and Maxillofacial Surgery between 1992 and 2009, were diagnosed with OSCC, and were available for long‐term follow‐up. The patients analyzed were treated with surgery, chemotherapy, or radiation therapy alone or in combination, and none of them were administered immune checkpoint inhibitors. Lymph node metastases were confirmed based on clinical findings or computed tomography (CT).

Analyses were performed using parameters, such as age, sex, site of tumor onset, stage, tumor–node–metastasis (TNM) classification, histological differentiation, and treatment modalities, of the included patients.

The tumor stage and TNM classification were according to the format defined by the Union for International Cancer Control (TNM Classification of Malignant Tumors, 7th Edition), and the degree of histological differentiation was classified into three grades (well, moderate, and poor), as defined by the World Health Organization Classification of Tumors, Pathology and Genetics of Head and Neck Tumors, 3rd Edition. Paraffin‐embedded sections obtained at the time of biopsy or surgical resection were used. This study was approved by the Ethics Committee of Aichi Medical University School of Medicine (accepted on June 3, 2014, 9–27) and conformed to the Declaration of Helsinki.

### 2.2 Immunohistochemistry

The specimens were fixed in formalin solution based on routine procedures for histopathology, and 4‐μm paraffin thin sections were prepared from paraffin‐embedded tissues. After deparaffinization, the sections were immersed in 10 mM citrate buffer and subjected to antigen retrieval using a microwave and blocking treatment to prevent the non‐specific protein binding. Sections were reacted with mouse monoclonal anti‐FoxP3 [236A/E7] (1:100, ab20034, Abcam, Cambridge, UK) and mouse monoclonal anti‐HPV (1:5, M3528, DaKo, Glostrup, Denmark) primary antibodies recognized non‐conformational internal linear epitope of major capsid protein of HPV overnight at 4°C. A horse anti‐mouse Ig secondary antibody (ImmPRESS™ REAGENT, VECTOR) was allowed to react for 1 h at room temperature, followed by color development with 3‐3′diaminobenzadine (DAB) (Wako Pure Chemical Industries Ltd., Osaka, Japan), counterstaining with hematoxylin, dehydration, mounting, and microscopy. Normal mouse serum instead of the primary antibody was used as the negative control.

### 2.3 Quantification of FoxP3 and HPV expression

Computerized image analysis systems consisting of an Olympus (BX53) light microscope (Olympus, Tokyo, Japan) and Olympus (DP21) charged‐couple device cameras were used to observe FoxP3‐ and HPV‐positive cells. In all 106 patients, immunohistochemically stained paraffin‐embedded sections were observed at higher magnification (400×). Assessments were performed by two independent observers (T.H. and K.Y.). One region was 0.09 mm^2^.

For FoxP3, the area with the highest number of positive cells was selected, and the number of positive cells was manually counted using enlarged images.

### 2.4 Statistical analysis

The JMP software (Ver. 13.2.1, SAS Institute Inc., NC, USA) was used for statistical analyses. FoxP3, HPV status and clinicopathological characteristics of OSCC were compared using the two‐sample independent t‐test or Wilcoxon's rank‐sum test for continuous variables and Fisher's exact test for categorical variables. Association of HPV status and location in oral cancer patients was assessed using logistic regression analysis and the *p*‐value was calculated based on the Wald statistics. Survival analyses were conducted for the outcomes of both 5‐year survival and overall survival via the Kaplan–Meier method, log‐lank test, and Cox regression analysis, with time to each outcome calculated from the date of diagnosis. An event was defined as recurrence in any form or death from any cause considering only the first event. Patients without events were censored at the date of last known follow‐up. Results for all analyses were only considered as statistically significant if *p*‐value was <0.05.

## 3 RESULTS

### 3.1 Association with clinicopathological indicators in target patients

The characteristics of study patients are shown in Table 1. Of the 106 patients included, 59 were men and 47 were women, with a mean age of 62.7 (20–93) years. The most common tumor site was the tongue in 53 patients, followed by the gingiva in 44.

**Table 1:** FoxP3 and HPV expression in OSCC and its association with clinicopathological indicators

Variable		FoxP3 low	FoxP3 high	*p*‐value	HPV negative	HPV positive	*p*‐value
*N*	*n* = 106	37 (35)	69 (65)		41 (39)	65 (61)	
Age, years (SD)	62.7 ± 16.1	60.6 ± 15.7	63.8 ± 16.2	0.34	63.0 ± 16.8	62.5 ± 15.7	0.89
Gender							
Male	59 (56)	21 (57)	38 (55)	1.00	22 (54)	37 (57)	0.84
Female	47 (44)	16 (43)	31 (45)		19 (46)	28 (43)	
Location							
Tongue	53 (50)	18 (49)	35 (51)	0.65	18 (44)	35 (54)	0.05^*^
Gingiva	44 (42)	17 (46)	27 (39)		16 (39)	28 (43)	
Others (lip, buccal, oral floor)	9 (8)	2 (5)	7 (10)		7 (17)	2 (3)	
Stage							
I	14 (13)	5 (13)	9 (13)	0.93	6 (15)	8 (12)	0.82
II	25 (24)	10 (27)	15 (22)		10 (24)	15 (23)	
III	41 (39)	14 (38)	27 (39)		17 (41)	24 (37)	
IV	26 (25)	8 (22)	18 (26)		8 (20)	18 (28)	
T classification							
1	16 (15)	6 (16)	10 (14)	0.95	6 (15)	10 (15)	0.72
2	37 (35)	14 (38)	23 (33)		17 (41)	20 (31)	
3	28 (26)	9 (24)	19 (28)		10 (24)	18 (28)	
4	25 (24)	8 (22)	17 (25)		8 (20)	17 (26)	
N classification							
0	65 (61)	27 (73)	38 (55)	0.09	25 (61)	40 (62)	1.00
1‐3	41 (39)	10 (27)	31 (45)		16 (39)	25 (38)	
M classification							
0	105 (99)	36 (97)	69 (100)	0.35	40 (98)	65 (100)	0.39
1	1 (1)	1 (3)	0 (0)		1 (2)	0 (0)	
Tumor grade							
Well	47 (44)	13 (35)	34 (49)	0.24	19 (46)	28 (43)	0.76
Mod	45 (42)	20 (54)	25 (36)		18 (44)	27 (42)	
Poor	14 (13)	4 (11)	10 (15)		4 (10)	10 (15)	
Treatment							
None	1 (1)	0 (0)	1 (1)	0.38	0 (0)	1 (2)	0.71
Surgery	13 (12)	6 (16)	7 (10)		6 (15)	7 (11)	
Chemotherapy+radiotherapy	33 (31)	14 (38)	19 (28)		14 (34)	19 (29)	
Surgery+radiotherapy+chemotherapy	59 (56)	17 (46)	42 (61)		21 (51)	38 (58)	
FOX‐P3							
Low		37 (35)	0 (0)		16 (39)	23 (35)	1.00
High		0 (0)	69 (65)		25 (61)	42 (65)	
HPV							
Negative		14 (38)	27 (39)	1.00	41 (39)	0 (0)	
Positive		23 (62)	42 (61)		0 (0)	65 (61)	

*Note:* *p*‐value by Student's *t*‐test or Wilcoxon's rank sum test for continuous variables and Fisher's exact test for categorical variables. ( ), the ratio of event (%).

*denotes a number that was significant in the statistical analysis.

By stage classification, there were 14 Stage I patients, 25 Stage II, 41 Stage III, and 26 Stage IV. For the TNM classification, there were 16 T1 stage patients, 37 were T2 stage, 28 were T3 stage, and 25 were T4 stage, with 41 patients who had nodal metastases that accounted for 39% of the entire population. There was one patient with distant metastases. Based on classifications according to the grade of histological differentiation, there were 47 patients with well‐differentiated, 45 with moderately differentiated, and 14 with poorly differentiated tumors. In head and neck cancer, the tongue is the most common site of onset, with slightly more men being affected in terms of the male to female ratio; the same tendency was observed in the patients in this study. Cervical lymph node metastases are generally identified in approximately 50% of patients at the time of diagnosis, and the prognoses of these metastatic patients are said to be poor (Kowalski et al., 2000; Zanoni et al., 2019); however, among the target patients in this study, the number of patients with lymph node metastases was comparatively lesser. Target patients were subjected to surgery, chemotherapy, or radiation therapy alone or in combination.

### 3.2 FoxP3 and HPV expression

**Figure 1:**
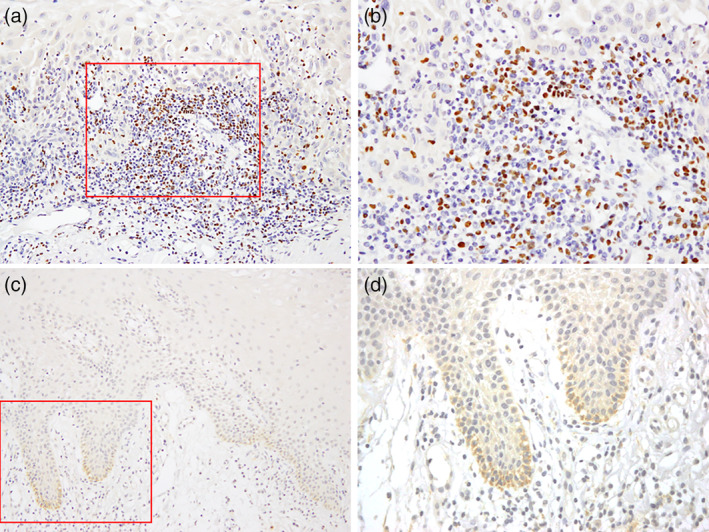
FoxP3 and HPV were detected using immunohistochemistry. (a, b) FoxP3 was stained in the nuclei of cells that were believed to be Tregs infiltrating the peritumoral stroma. (c, d) HPV antigens were stained in cells forming the base membrane of cancer tissues. Magnification 200× (a, c); 400× (b, d)

In this study, the lymphocytes were primarily in the periphery of the tumor cells. This infiltration only in the vicinity of the tumor cells is equivalent to “excluded” in the classification of the tumor immune microenvironment (TIME) by Binnewies et al. (2018). FoxP3 was clearly stained in the nuclei of lymphocytes infiltrating the tumor tissue. FoxP3‐positive cells also infiltrated to the stroma area in most cases, with a few cases invading the tumor area. A typical example is shown in Figure 1(a,b). If the number of <10 positives in the observation area is considered negative, 98 of the 106 cases included in this study (92%) were positive and showed a high frequency of FoxP3‐positive cells infiltrating the tumor tissue.

Among the target patients, 65 (61%) were confirmed to have HPV‐stained plasma membranes centered on the basement membranes of cancerous cells and classified as HPV‐positive. The remaining 39 patients (41%) were classified as HPV‐negative (Figure 1(c,d)). There was a significantly higher HPV infection rate in the gingiva and tongue compared that at other sites (*p* = 0.04, *p* = 0.02) (Table 2).

**Table 2:** Association between HPV expression and sites of infection in the oral cavity

HPV positive			Odds ratio (95%CI)		
Others (lip, buccal, oral floor) (o)	Gingiva (g)	Tongue (t)	o vs g	o vs t	g vs t
7 (17)	16 (39)	18 (44)	6.13 (1.13, 33.10)	6.81 (1.27, 36.193)	1.11 (0.48, 2.57)
			*p* = 0.04*	*p* = 0.02*	*p* = 0.81

*Note:* The number of patients; ( ), the ratio of event (%); *p*, *p*‐value by Wald method.

*denotes a number that was significant in the statistical analysis.

### 3.3 Associations between FoxP3 and HPV expression and survival prognoses

**Figure 2:**
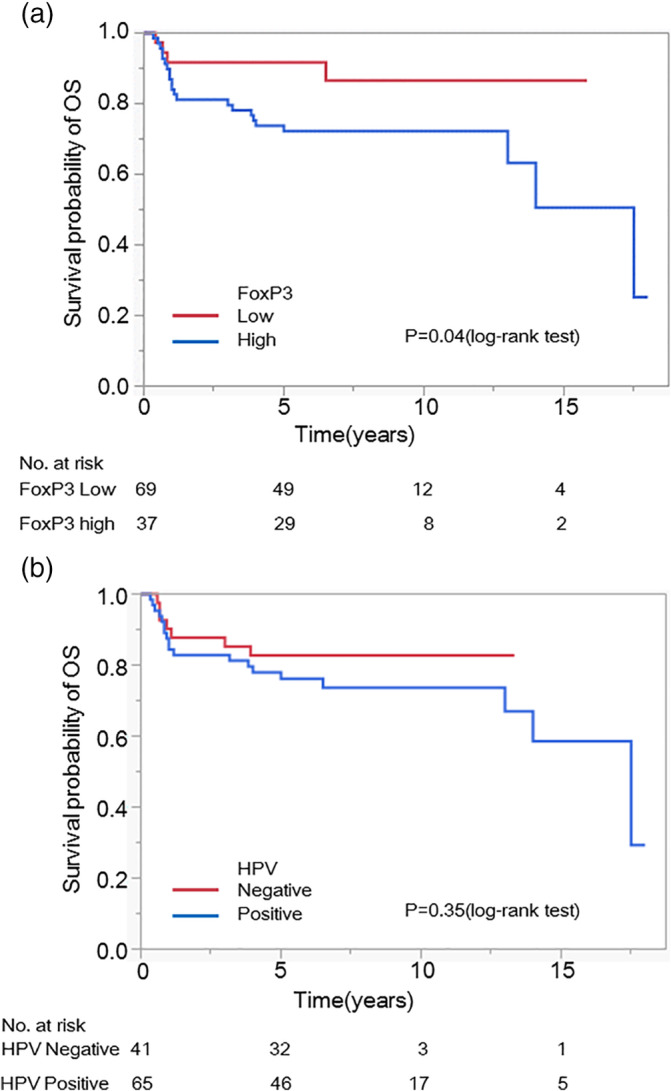
Association between FoxP3 and HPV expression and survival prognoses. (a) A total of 106 patients were classified using immunohistochemistry as FoxP3 high or low, and overall survival was compared. A significant worsening of survival was observed in FoxP3 high patients (*p* = 0.04). (b) The same patients were also classified as HPV‐positive or ‐negative, and overall survival was compared; however, the differences were not significant (*p* = 0.35)

The correlation between FoxP3 expression and prognosis was investigated, and the number of FoxP3‐positive cells with >50 in cases, the prognosis was worse than in cases with <50 units (*p* < 0.05) (Figure 2(a)). Therefore, cases with >50 FoxP3‐positive cells were classified as “FoxP3 high” cases, and those with <50 cells were classified as “FoxP3 low” cases. It was further examined as a case study.

Comparisons of overall and 5‐year survival in FoxP3 high and FoxP3 low patients with OSCC showed significant worsening of both survival parameters in FoxP3 high patients (*p* = 0.04) (Figure 2(a)). A 2.90‐fold increased risk of mortality [95% confidence interval (CI): 1.10–9.93] was also shown in FoxP3 high patients compared with that in FoxP3 low patients (Table 3).

**Table 3:** Association of FoxP3 expression (high and low) and HPV infection status (positive and negative) with 5‐year survival/hazard ratio

Outcome	5‐Year survival rate for FoxP3 low	5‐Year survival rate for FoxP3 high	*p*	Unadjusted HR (95%CI)
OS	91.7	72.3	0.04*	2.90 (1.10, 9.93)

Outcome	5‐Year survival rate for HPV negative	5‐Year survival rate for HPV positive	*p*	Unadjusted HR (95%CI)
OS	76.2	82.7	0.35	1.52 (0.65, 3.94)

*Note:* *p*‐value by log‐rank test.

Abbreviation: HR, hazard ratio.

*denotes a number that was significant in the statistical analysis.

Comparisons of overall and 5‐year survival in HPV‐positive and ‐negative patients with OSCC showed a tendency toward a slightly worse prognosis in HPV‐positive patients; however, a strong correlation was not observed (*p* = 0.35) (Figure 2(b); Table 3).

### 3.4 Associations between FoxP3 and HPV expression and clinicopathological indicators

The associations between FoxP3 and HPV expression and clinicopathological indicators are shown in Table 1. There were no significant associations between the expression of FoxP3 and age, sex, site of tumor onset, stage, T classification, distant metastases, histological differentiation, or treatment. However, although a significant association between the expression of FoxP3 and the presence of lymph node metastases was not observed (*p* = 0.09), the expression of FoxP3 was considered to have had some effect on lymph node metastases.

There were no significant associations between HPV expression and age, sex, stage, T classification, distant metastases, histological differentiation, and treatment. However, a significant association between HPV infection and the site of tumor onset was observed (*p* = 0.05), indicating some influence between site‐specific HPV infection and tumors (Table 1).

## 4 DISCUSSION

Tregs play a critical role in immune homeostasis by maintaining immune self‐tolerance through the suppression of excessive immune responses. Meanwhile, Tregs suppress antitumor immune responses and may also contribute to the onset and exacerbation of malignancies (Nishikawa & Sakaguchi, 2010). In several carcinomas, including gastric, esophageal, and renal cell cancers, a large number of Tregs are commonly found in the peripheral blood, primary tumors, metastases, and lymph nodes of patients. Negative correlations between the numbers of Tregs and tumor grade or patient prognoses have been reported (Kono et al., 2006; Liotta et al., 2011). Moreover, a potential increase in the number of Tregs in carcinomatous ascites has also been reported (Liotta et al., 2011). However, in colorectal cancer, an increased number of Tregs has been reported to be a predictor of favorable outcomes (Salama et al., 2009), and in head and neck cancer, Tregs reportedly inhibit inflammation that promotes tumor progression (Badoual et al., 2006). These previous findings highlight the lack of concordant views on Treg's roles in cancer. A recent review reported on Tregs in oral and oropharyngeal cancers (O'Higgins et al., 2018). Forty‐five articles were reviewed, of which 39 studies were conducted in humans. In these articles, Foxp3 was the most commonly used marker for Tregs. Twenty‐five of these articles suggested that an increase in the number of Tregs in the tumor microenvironment and peripheral blood was associated with worse prognoses, whereas nine reported that this increase was associated with more favorable prognoses, particularly in the setting of HPV infection.

Thus, in this study, by showing the presence of FoxP3+ Tregs and HPV in patients with OSCC in Japan, part of the immune response localized to the area of the tumor was clarified, and the associations with survival prognoses and clinicopathological indicators were evaluated. In the evaluation of the number of FoxP3‐positive cells, the cutoff value was tested with various numbers of positive cells, and the two groups were divided into two groups with 50 cells as the number that could be significantly classified. For HPV, the analysis was divided into two groups: positive and negative by staining with anti‐HPV antibodies.

Upon evaluation of the association between the presence of FoxP3+ Tregs in OSCC and survival prognosis, a tendency of the survival prognosis to deteriorate was shown in FoxP3+ Treg High patients, demonstrating an elevated mortality risk. In this study, the increase in the number of Tregs in OSCC was shown to suppress antitumor immune responses and contribute to the onset and exacerbation of malignancies; thus, evidence supporting the possibility of a new therapy for OSCC that improves survival prognoses by targeting Tregs was provided. Defining the causes of quantitative differences in induced FoxP3+ Tregs and immune responses in the tumor microenvironment is a challenge for the future.

In recent years, studies on the association between the onset of head and neck cancer and HPV infection have been conducted, and various results have been shown (Elango et al., 2011; Gillison, 2000; Lee et al., 2012; Simonato et al., 2008). In particular, the association between oropharyngeal cancer and HPV infection is strongly suggested, and therapeutic efficacy and favorable prognoses have been reported for oropharyngeal cancer that may be caused by HPV infection (Gillison, 2000). Conversely, among head and neck cancers, HPV infection reportedly does not play a major role in the progression of oral floor cancer (Simonato et al., 2008), is positively correlated with tongue cancers (Elango et al., 2011), increases the risk for distant metastases, and reduces the survival rate (Lee et al., 2012); thus, the clinical significance of HPV infection remains unclear (Lai et al., 2017). The evaluation of HPV‐infected tumors in this study showed that 60% of patients with OSCC were HPV‐positive, whereas the study by Lajer and Buchwald (2010) reported that HPV positivity ranged from 0% to 74% in patients with OSCC, indicating variations among the patients studied.

Upon evaluating the associations between the presence of FoxP3+ Tregs and clinicopathological indices in HPV‐infected patients with OSCC, no significant associations were found. However, when the prognoses of HPV‐positive and ‐negative patients with OSCC were evaluated, unlike in oropharyngeal carcinoma, they tended to be slightly worse in HPV‐positive patients with OSCC. In other words, this suggested that HPV infection in patients with OSCC, unlike oropharyngeal carcinoma, did not result in more favorable outcomes. This result is a step forward toward unveiling the association between HPV infection and prognoses in head and neck cancers other than oropharyngeal cancer. As indicated by Andersen et al. (Andersen et al., 2014), unlike the mucosal epithelia of other common sites of HPV16 infection (uterine cervix and anogenital regions), there is an anatomical difference between oropharyngeal carcinoma and the HPV‐infected mucosa in OSCC (i.e., whether lymphoid tissue is also present in the submucosa), which may have resulted in differences in immune responses in the tumor microenvironment and impacted prognosis. Meanwhile, oropharyngeal carcinoma has been reported to have a favorable prognosis depending on the extent of infiltration of tumor‐infiltrating lymphocytes (TILs), and there are contradictory reports regarding whether the extent of infiltration of TILs has an association with the HPV infection status (Nordfors et al., 2013; Oguejiofor et al., 2015; Wansom et al., 2012). Thus, a future challenge will be to elaborate the immunological milieu, such as the site of onset, HPV status, the expression of immune checkpoint molecules, and infiltrating immune‐related cells in the local tumor site, in order to clarify the treatment selection for OSCC.

In conclusion, the findings of this study revealed that the increase in the number of Tregs, which originally play a critical role in immune homeostasis by maintaining immune self‐tolerance via the suppression of excessive immune responses, was associated with the deterioration of prognoses in OSCC. These results suggest the potential for new OSCC therapies that target Tregs. Unlike in oropharyngeal cancers, HPV‐positive patients with OSCC tended to experience slightly worse outcomes; this was attributed to differences in immune responses in the tumor microenvironment due to anatomical differences in the mucosa infected with HPV.

## CONFLICT OF INTEREST

All authors declare that there is no conflict of interest.

## AUTHOR CONTRIBUTIONS

Tomio Hayashi and Kazuhiro Yoshikawa were responsible for the design of the study, the histological evaluations, the data analysis, and the drafting of the article. Masahiko Gosho took part in the data analysis. Susumu Suzuki, Ryuzo Ueda, and Yoshiaki Kazaoka participated in discussions during the experiments and writing of the article and offered valuable advice.

## Data Availability

The data that support the findings of this study are available from the corresponding author upon reasonable request.
